# Synthetized Potato Starch—A New Eco Sizing Agent for Cotton Yarns

**DOI:** 10.3390/polym11050908

**Published:** 2019-05-20

**Authors:** Stana Kovačević, Ivana Schwarz, Suzana Đorđević, Dragan Đorđević

**Affiliations:** 1 Department of Textile Design and Management, Faculty of Textile Technology, University of Zagreb, Prilaz baruna Filipovića 28a, 10000 Zagreb, Croatia; stana.kovacevic@ttf.hr; 2 Textile Department, Higher Technological and Artistic Professional School Leskovac, Vilema Pusmana 17, 16000 Leskovac, Serbia; szn971@yahoo.com; 3 Textile Department, Faculty of Technology, University of Nis, Bulevar Oslobodjenja 124, 16000 Leskovac, Serbia; drdrag64@yahoo.com

**Keywords:** synthesized biopolymer materials, potato starch, hydrolysis, grafting, cotton yarn, size pick-up, breaking force, abrasion resistance, yarn hairiness, desizing

## Abstract

The objective of this research was to verify the feasibility of the use of newly synthesized biopolymer materials for sizing cotton yarns based on the basic principles of chemical modification. Research included acid hydrolysis of potato starch up to controlled molar masses together with graft-polymerization and methacrylic acid onto hydrolyzed starch to improve hydrophilicity and solubility, to increase the capability of film forming, to increase adhesive potential and to avoid retrogradation phenomena. Research objectives were primarily focused on finding an appropriate, environmentally-friendly and productive sizing agent for cotton yarns via the analysis and systematization of a large number of synthesis methods in conjunction with the characterization and properties of graft-copolymers. The research results showed that potassium persulfate initiator was most efficient in grafting of methacrylic acid onto hydrolyzed starch, while azobisisobutyronitrile (AIBIN) initiator was most efficient in grafting of acrylic acid (AC). FTIR analysis confirmed that new and efficient products for sizing cotton yarns from synthetized potato starch were obtained. Research on rheological properties of copolymers shows a higher viscosity of grafted products indicating the good stability of potential starches. Ecological improvements have been established through high desizing degree as well as improvements in physical-mechanical properties of yarn, abrasion resistance and decrease in yarn surface hairiness were noticed. The use of new derivatives of potato starch, especially of hydrolyzed starch grafted with methacrylic acid (MAA), potassium persulfate (KPS) as initiator, was confirmed. Anova statistical analysis determined the influence of the entire sizing process on individual yarn parameters.

## 1. Introduction

### 1.1. Sizing Process

Sizing is a technological process of wet processing of warp yarns to obtain additional properties which are necessary for weaving. The sizing process is nowadays more aimed towards the development and use of natural-based sizing agents. The sizing process is one of the most important and expensive phases in fabric manufacturing; so its contribution in its development and improvement is reflected through the development and automation of sizing machines and the introduction of new, more efficient and more cost effective sizing agents. By using synthetic polymer products, sizing gains significance as a phase in which yarn properties can be greatly optimized, which significantly increases the productivity of the weaving mill and fabric quality. The basic objective of sizing is that the use of the appropriate polymer capable of forming a protective layer on the yarn provides the durability of warp threads and their minimum deformation during short and strong cyclic stresses in the weaving process [1,2,3,4,5,6,7].

Sizing efficacy does not depend only on the adhesion between the applied sizing agent and the yarn, but also on the ability to form a film, on rheological properties of the size, physical-chemical yarn properties, as well as on the technological parameters in the sizing process. Furthermore, it is necessary to completely remove the sizing agent from the fabric after the weaving process in an environmentally friendly manner. Under production conditions it often happens that inadequate sizing agents are used, especially inadequate recipes.

**Figure 1:**
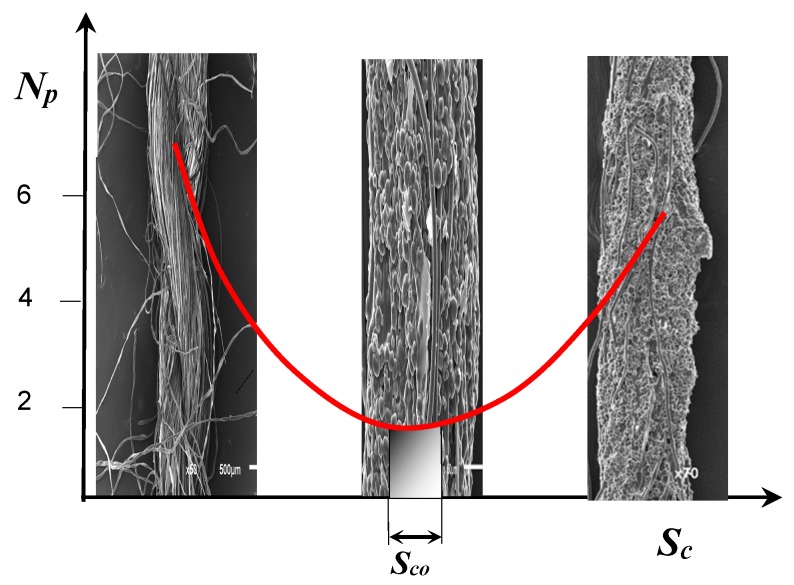
Effect of size pick-up on the number of breakages, with highlighted area of optimal size pick-up; Np—number of breakages of warp threads per machine and per hour, Sc—size pick-up, Sco—optimum size pick-up (%).

The optimization of the proportion and application of sizing agents to the warp threads is of the utmost importance for the entire weaving process in order to achieve the planned machine utilization and the quality of the finished fabric. Optimization of size pick-up is one of the biggest problem in the sizing process even today. The purpose of sizing is to reduce the number of warp thread breakages to a minimum. Observing the curve of the warp threads breakage number (Figure 1) there is noticeably a drastic decrease with the increase of size pick-up (Sc) to a certain minimum indicating optimal size pick-up (Sco). Further increase causes an excessive amount of size pick-up, which makes threads glued to each other causing difficult separation in the subsequent process. The consequence of this is manifested through yarn unevenness and increased number of breakages during the weaving process. Finding the right recipe that will provide the lowest number of breakages on the weaving machine is the main goal of any technologist [8,9,10,11,12].

The question is posed whether sizing is unconditionally necessary, because it practically represents an ecological and economic burden in fabric manufacturing. The complexity of the sizing process is reflected in a number of parameters related to the properties of sizing agents, used yarns and sizing and weaving machines which is always a challenge and is a significant field of research.

### 1.2. Sizing Agents

Based on the chemical composition, the sizing agents used nowadays can be divided into the following groups: starches, carboxymethylcellulose, polyvinyl alcohols, polyacrylates and polyester resins. From the group of starches, potato is one of the most commonly used. It disadvantage is that a small drop in temperature suddenly changes the rheological properties of size, and this results in retrogradation, which causes difficult control of the degree of sizing.

Starch properties depend on the relationship between amylose and amylopectin, but also on other constituents found in a starch granule, such as phosphates, lipids, phospholipids, etc. However, amylopectin, as the main starch ingredient, exerts a dominant influence on starch properties. The lateral chain length in amylopectin affects clustering, retrogradation and starch properties in the solution. In cold water starch swells, but does not dissolve. Starch granules disintegrate only at elevated temperatures, i.e., dissolving happens whereby amorphous amylose is released first which forms a three-dimensional network outside starch granules and inhibits further swelling of starch granules. Besides amylose, the present lipids also inhibit swelling. Although amylose makes only a slight contribution to the viscosity of the starch solution after granule disintegration, when cooling the viscosity of the starch solution increases with increasing amylose content, which is explained with amylose crystallization and gel structure formation. It is known that, if starch solution is allowed to stand, starch retrogradation occurs, which is one of bad indicators for commercial use as a sizing agent. An increase in the concentration and a decrease in the solution temperature accelerate retrogradation, while very short chains (degree of polymerization 6–9) suppress starch retrogradation [13,14,15,16].

In order to allow recycling using ultra-filtration, sizing agents must have the following characteristics: water solubility, mechanical and thermal stability, biological resistance, good washing performance, low average viscosity [17,18,19].

Starch is mostly commercially applied in sizing cotton warp yarns, although with considerable shortcomings: (a) molecule size which limits the penetration into the yarn, (b) instability of size viscosity due to temperature changes during cooking until preparation and sizing process, (c) film rigidity (in particular in the absence of a high-quality lubricant), (d) sensitivity to microorganisms (decay, degradation). Modification of natural starches is done just to eliminate the above mentioned shortcomings and to increase their usability for industrial applications [20,21,22,23].

Hydrolysis of potato starch and vinyl monomers—acrylic (AA) and methacrylic acid (MAA) by using various initiators (azobisizobutyronitrile (AIBN), potassium persulfate (KPS) and benzoyl peroxide (BPO) was performed. The use of AA monomer in the process of grafting natural starch was found in textile finishing, but not in yarn sizing. Also, the absence of research of starch MAA grafting for use in sizing was crucial for their choice, assuming the behavior in the practice of the modified size film on the yarn, taking into consideration the presence of polar and non-polar parts in the structure of sizing agents. Hydrolysis of starch was carried out in order to reduce molar mass, which implies easier solubility without bulky agglomerates in the size, easier penetration into the yarn structure and later easier removal from the fabric. Monomers were grafted onto shorter starch macromolecules, creating lateral shorter or longer branches on the main chain, thus forming a product for a more efficient use in yarn processing. A variation of the initiator was performed in order to form grafted monomers more efficiently on the starch and because of the differences of bonded ones on longer or shorter lateral chains and/or denser packing of lateral chains. Later, that can greatly affect size rheology and the behavior of the film on the impregnated yarn [24,25].

The optimization of the technological sizing process conditions refers to the definition of the particular parameters of the laboratory sizing machine: thread tension between the bobbin creel and the size box; temperature of the sizing agent in the size box; sizing speed—passage of the threads through the size box; pressure of the immersion and squeezing rollers; temperature of the cylinders of the contact dryer, size circulation and exit moisture.

Cotton yarns of different structures (single and double) were sized using different size recipes in order to find the optimal ones, which would, in practical terms, achieve the most efficient results on the weaving machine. During the research the best conditions of grafting, temperature-time regime, type of initiator and monomer were defined [26,27,28]. Furthermore, the research objectives establish their foothold in the fact that new agents require a new approach and a new technological organization for the use, i.e., in the sizing process of cotton yarns with new sizing agents. Therefore, part of the objectives includes optimizing the conditions of the technological sizing process on a laboratory sizing machine adapted to the conditions of the industrial sizing conditions. The objectives of this research relate primarily to the establishment of adequate, environmentally friendly and productive systems for warp sizing via the analysis and systematization of a large number of synthesis procedures in conjunction with the characterization and properties of graft copolymers.

## 2. Materials and Methods

### 2.1. Yarns

For the purposes of this research 100% ring spun single cotton yarns with counts of 20 tex and 30 tex (700 twists/m) and ply yarns with counts of 20 × 2 tex and 30 × 2 tex (324 twists/m) were used. Egyptian cotton fibers with lengths 27–28 mm and average diameter of 4.5 μm were spun into a yarn. The yarn was spun and plied by the Textile Company Bimtex, Leskovac, Serbia.

### 2.2. Sizing Agents

For the purpose of this research the following active products were used: potato starch (25 wt.% amylose, “Jabuka” Starch Industry, Pančevo, Serbia), hydrochloric acid—HCl (Centrohem, Stara Pazova, Serbia), ethyl alcohol (ethanol)—C_2_H_5_OH (Reahem, Novi Sad, Serbia) and sodium carbonate—Na_2_CO_3_ (LG Hemija, Belgrade, Serbia).

The following initiators in the grafting process were used: azobisisobutyronitrile (AIBN), i.e., (2,2′-azobis(2-methylpropionitrile)) 99% (Merck KGaA, Darmstadt, Germany), potassium persulfate (KPS) K_2_S_2_O_8_ (Centrohem, Stara Pazova, Serbia) and benzoyl peroxide (BPO) (C_6_H_5_CO)_2_O_2_ (Merck KGaA, Darmstadt, Germany). As monomers in the grafting process were used: acrylic acid (AA) C_3_H_4_O_2_ (Merck KGaA, Darmstadt, Germany) and methacrylic acid (MAA) C_4_H_6_O_2_ (Merck KGaA, Darmstadt, Germany).

Table 1 lists the designations of the copolymers obtained after graft copolymerization onto starch. When copolymerizing with AIBN, BPO and AP initiators, the grafted branches are bonded with the starch skeleton over oxygen, because the graft copolymerization is initiated by the release of the active hydrogen from starch hydroxyl [29].

The structures obtained by copolymerization are very complex, which is confirmed in many works with polysaccharides. Most commonly, graft polymerization is brought about by free radical addition polymerization. Even though a large number of works on graft copolymerization has been published, there is not a comprehensive understanding of the grafting process yet. The grafting schemes in Table 1 are a reflection of the literature data and similar research [30,31,32,33,34,35,36]. Considering the large amount of nonreducing diol groups along the polysaccharides chains, both C1-C2 and C2-C3 glycol groups are the predominant sites for initiation of graft copolymerization. Trans glycol groups and C6-hydroxyl groups have negligible reactivities [37].

### 2.3. Sizing on the Laboratory Sizing Machine

**Figure 2:**
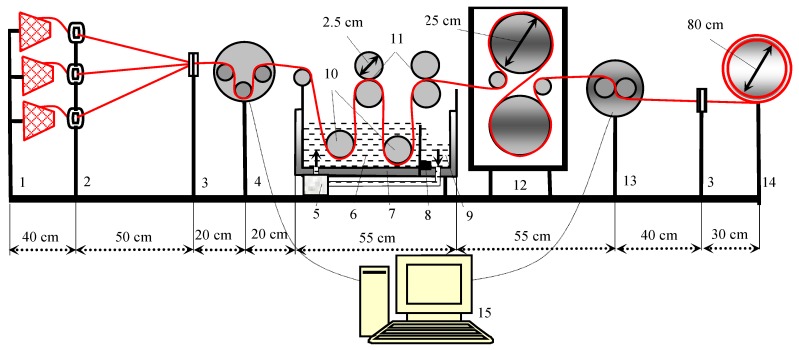
Laboratory sizing machine; 1—creel for cross wound bobbins, 2—thread tensioners, 3—comb, 4—tensiometer, 5—pump, 6—size box, 7—double wall of the size box, 8—thermostat, 9—prewetting size box, 10—immersion rollers, 11—size squeezing rollers, 12—contact dryer, 13—moisture measurement unit, 14—yarn winder, 15—computer with A/D converter for data storage.

The yarn sizing process was carried out on an innovative laboratory sizing machine adapted to industrial conditions (University of Zagreb Faculty of Textile Technology, Zagreb, Croatia, Figure 2). The laboratory sizing machine can adjust sizing speed, thread tension, pressure of squeezing the excess size paste from the yarn on the last pair of the squeezing rollers and also the adjustment of the size pick-up; it enables the circulation of the size from the pre-box into the size box and vice versa, maintains the size paste temperature by heating the walls of the size box and enables the adjustment of the drying temperature in the contact dryer.

During the sizing process percentages of the copolymer solution in the water (concentration) for the single yarn were: 5%, 10% and 15%, and for the ply yarn: 1%, 3% and 5%, adapted to industrial conditions. In the weaving process warp threads are strained and subjected to dynamic stresses, resulting in their deformation and breakage. It is not necessary to size the ply yarn with the amount of starch as a single yarn because ply yarns mostly have satisfactory tenacity properties. In that case, it is only sufficient to connect the fibers on the yarn surface, which is conducted by using lower concentrates of starch. The size was prepared by mixing sizing agents in cold distilled water. After grinding, the bath was gradually heated with intense mixing up to 80 °C during 30 min and then 5 min at a temperature of 95 °C. Afterwards the size paste is ready for sizing, and the following conditions are adapted before sizing:

- Thread tension in the entry into the size box: 42 cN
- Size temperature: 85 °C
- Sizing speed: 3 m/min
- Pressure on the last pair of size paste squeezing rollers: 1.5 N/cm^2^
- Drying temperature: 110–130 °C
- Exit moisture: 5.5%.

### 2.4. Tested Parameters, Devices and Standards

To conduct an overall analysis, tests on unprocessed and processed samples were carried out according to standardized methods:

- Determination of average values of molecular weights and the distribution of copolymer molar weights (M_n_, M_w_, M_z_, D) was conducted by gel permeable chromatograph GPC Agilent 1100 Series, Agilent Technologies, Santa Clara, CA, USA, using a differential refractometer (RID detector) of 1200 Series, Agilent Technologies, USA, as a detector. M_n_ represents the number average molecular weight calculated from the number of macromolecules of the polymer, while M_w_ represents weight average molecular weight calculated on the basis mass of these macromolecules. M_z_ is z-average molecular weight that also depends on the size and weight of molecules. For polymers characterization it is essential to determine the polydispersity index D and to define the degree of polymer inequalities. Dispersion is a measure of molecular weight distribution or the degree of polymerization, which is for uniform polymer D = 1.
- Breaking force and elongation at break of the yarn tested on a Textechno tensile tester, model Statimat M (Textechno H. Stein GmbH & Co. KG, Moenchengladbach, Germany), according to standard ISO 2062.
- Unevenness testing carried out on a Keisokki Evenness Tester Model 80, type B (Keisokki Kogyo Co. Ltd., Amagasaki, Japan) according to ISO 16549. The criteria selected for yarn testing are: recording thick places of the yarn over 50% of the average thickness, recording thin places of the yarn under 50% of the average yarn thickness and recording yarn slubs (neps, knots) of the yarn over 200% of the average yarn thickness.
- Yarn hairiness tested on a Zweigle G 565 (Uster Technologies AG, Uster, Switzerland) instrument for yarn hairiness testing according to standard ASTM D 5674-01.
- Yarn twist was determined using a MesdanLab Twist Tester (Mesdan, Salo, Italy) according standard ISO 17202.
- Abrasion resistance of the yarn was measured on a Zweigle G551 yarn abrasion tester Desizing was conducted using ultrasound and washing off process. Ultrasound desizing process was performed in a Ultrasonic Laboratory Reactor URS 1000 (AlliedSignal ELAC Nautik GmbH, Kiel, Germany), with the frequency of applied ultrasound oscillations of 40 kHz, using 150 W, at 60 °C for 30 min. Washing off process was performed using washing machine Ahiba Linitest (Datacolor, Lawrenceville, GA, USA), by washing agent Lavan NH, Textilcolor AG, Sevelen, Switzerland (2 g/L concentration), at 60 °C for 30 min, followed by abundant water wash and air drying.
- The FTIR analysis of samples—potassium bromide technique, spectrophotometer BOMEM Hartmann & Braun MB-Series (ABB group, Zurich, Switzerland) in the range of wavelengths 4000–400 cm^−1^ was conducted.
- HPLC (high pressure liquid chromatography) method was used to investigate the residual amounts of the unreacted monomer in the copolymer. Tests were carried out on the device Agilent Series 1100 HPLC with a diode-array detector, DAD 1200 Series (Agilent Technologies, Santa Clara, CA, USA). The detection wave length is 205 nm. The ZORBAX Eclipse XDB-C18 column, 4.6 × 250 mm, 5 μm was used. The effluent is methanol, the flow rate is 1 mL/min, the column is thermostated at 25 °C, the injected volume is 20 μL. Monomer standards were prepared by single weighing of the sample with diluted methanol.
- Scanning Electron Microscopy (SEM) was performed on a JEOL JSM–6610 LV microscope (JEOL Ltd., Tokyo, Japan).
- Size pick-up (D) on the yarn was determined using the mass technique according to the equation:
  (1) $$D=\frac{m_{1}-m_{0}}{m_{0}}\;\times \;100\;\left(\%\right)$$
  where: *m*_1_—yarn mass after sizing (g); *m*_0_—yarn mass before sizing (g);
- Apparent viscosity on the rotational viscometer “Visco Basic Plus” (Fungilab S.A., Barcelona, Spain). The spindle speed of the viscometer is 60°/min, time: 15 min to 40 °C, 20 min to 60 °C and 30 min to 85 °C.
- Viscosity stability (*Vs*) according to the equation [38]:
  (2) $$V_{s}=\left(1-\frac{V_{max}-V_{min}}{V}\right)\;\times \;100$$
  where: *V*—apparent viscosity of size, *V_max_* and *V_min_*—maximum and minimum of the measured viscosity over a period of 7 h.
- Statistical analysis was performed using OriginPro software 9.3. (OriginLab Corporation, Northampton, MA, USA) Anova: Analysis of Multiple Linear Regression (Multiple Regression), linear linkage of key variables and their predictors, Levene test, Scheffe test, *F*-test, *t*-test and determination coefficient R were methods used for statistical analysis of tested results.

## 3. Results and Discussion

### 3.1. Size Modification

For the purposes of this research potato starch was used for hydrolysis and afterwards for grafting of various monomers. Starch hydrolysis was carried out to reduce molar weight, to increase the solubility and availability of active centers for chemical modification.

Table 2 shows the results of copolymers molar weights as well as the degree of polydispersity. In all tested samples M_w_ (weight average molecular weight) was higher than M_n_ (number average molecular weight), which confirms polydisperse systems, respecting the rule that the greater difference between the M_w_ and M_n_ is, the systems is more polydispersonal. It is known that all the synthetic polymers are polydispersed and the valid order for them is: M_z_ > M_w_ > M_n_ [39]. Less waste of the results shows the samples of hydrolysed grafted starch of the smallest molar weights with elution time of 4.3 to 6.5 min. Greater molar weight of grafted samples with eluting time of 1.6–4.2 min yields a lower uniformity.

Graft copolymerization was carried out whereby monomers were grafted onto shorter starch macromolecules, forming lateral branches on the main chain resulting in a product, which can be more efficiently used as a sizing agent. By increasing the index of polydispersity, the presence of chain contents with lower molar weight values is greater, whose mobility is also greater (for example sample HS-AA-KPS). Shorter chains are easily movable in the solution and serves as a “lubricator” (plasticizers) during movement of a longer chain or their aggregates [40]. A variation of the monomer, but also of the initiator was carried out primarily because of the more efficient grafting of the monomer onto starch, but also because of the diversities related to shorter or longer lateral chains, and their looser or tighter packing. 

Characterization of all grafted monomers onto hydrolyzed starch was performed based on the following equation:

– yield of starch hydrolysis (Ysh):
  (3) $$\mathrm{Ysh}=\frac{W_{1}}{W_{0}}\;\times \;100\;\left(\%\right)$$
– graft yield (Yg):
  (4) $$\mathrm{Yg}=\frac{W_{2}-\;W_{1}}{W_{1}}\;\times \;100\;\left(\%\right)$$
– grafting percentage (Pg):
  (5) $$\mathrm{Pg}=\frac{W_{2}}{W_{1}+\;W_{3}}\;\times \;100\;\left(\%\right)$$
– percentage of graft efficacy (Pge):
  (6) $$\mathrm{Pge}=\frac{W_{2}-\;W_{1}}{W_{3-\;W_{4}}}\;\times \;100\;\left(\%\right)$$
– conversion of monomer to polymer (Cmp):
  (7) $$\mathrm{Cmp}=\frac{W_{3}-\;W_{4}}{W_{3}}\;\times \;100\;\left(\%\right)$$
  where: *W*_1_—mass of hydrolysed starch, *W*_0_—mass of natural screed, *W*_2_—mass of grafted starch (gravimetric method), *W*_3_—mass of used monomer, *W*_4_—mass of residual monomer.

Table 3 reveal several parameters that show the hydrolysis efficacy and the performance of monomer grafting onto starch depending on the initiator type and FTIR spectra of the hydrolyzed and grafted starch. FTIR spectra of the grafted samples the most efficient associated initiator such as: AIBN initiator in grafting of acrylic acid onto starch, while KPS initiator is the best in grafting of methacrylic acid onto starch. The FTIR spectra of hydrolyzed potato starch are very similar, and slight differences stem from the origin of starch. According to the spectra of these starches, a broad peak of 3395 cm^−1^ is observed, which stems from O–H valence vibrations as well as a smaller peak at 2925 cm^−1^ which is attributed to the C–H valence vibration. Wave numbers at about 1153, 1078 and 1023 cm^−1^ describe C–O–C stretching (triplet of starch) and the maximum band at about 1638 cm^−1^ originates from water molecules [41,42].

Potato starch grafted with AA—in the spectrum characteristic bands appear which originate from polyacrylic acid. The carboxylic acid group is detected through the absorption of the carbonyl C=O group at about 1733 cm^−1^ (C=O stretching). If it is a carboxylate anion, there are two ν (C=O) maximums, one of which is at about 1637 cm^−1^ ν_as_ (C=O) and the other at about 1420 cm^−1^ ν_s_ (C=O). Bands at about 1458 and 1375 cm^−1^ indicate deformation vibrations of the methyl group, δ_as_ (CH_3_) and δ_s_ (CH_3_), respectively [43,44,45].

The confirmation of grafting of MA onto the hydrolyzed potato starch can be found in new bands in the FTIR spectrum. The absorption with a maximum at about 1716 cm^−1^ originates from valence vibrations C=O. Also, bands coming from valence of the O–H group at about 3410 cm^−1^ are formed, valence vibrations of CH_2_ group at about 2927 cm^−1^ and deformation vibrations of CH_3_ in the plane having a maximum at 1374 cm^−1^ [46].

Table 3 show the single parameters which show hydrolysis efficacy and performance of grafting of monomers onto starch in dependence of the initiator type. AIBN initiator was very efficient in grafting of acrylic acid (AA) onto starch, considering the results of yield, percentage and efficacy of grafting. Grafting in the presence of KPS, as initiator, has slightly lower values of the tested parameters, but a higher value of the conversion of monomer to polymer, which, when analyzing other parameters, indicates the fact that a higher conversion can cause an unproductive reaction—formation of more homopolymers and less grafted polymer. Initiator potassium persulfate (KPS) is very efficient in grafting of methacryl acid (MAA) onto starch, taking into consideration the results of yield, percentage and efficacy of grafting. Grafting in the presence of AIBN and BPO has slightly higher values of the conversion of monomer to polymer, indicating that a higher conversion can cause an unproductive reaction—formation of more homopolymers, and fewer graft polymers.

Chromatography is a high-performance liquid method (HPLC) used to separate liquid mixtures. According to the obtained HPLC results of hydrolyzed starch, it is possible to determine the difference between graft products and initiators. Table 4 shows the HPLC chromatogram (RID signal) of the aqueous extract of a synthesized sample of starch copolymer, depending on the type of the used initiators, which shows that a corresponding response of the chromatographic signal of the copolymer appears under the given conditions in dependence of the initiators employed. Starch copolymer-AA grafted with AIBN and BP initiators has the highest intensity in the chromatogram with the greatest area.

### 3.2. Rheological Properties of Copolymers

The viscosity of the sizing liquor, which is also defined as its flow, mostly depends on the time of left standing unused, on circulation and temperature. During the preparation of the solution it is noted that at lower concentrations of up to 3%, the solution of hydrolyzed potato starch behaves almost like a Newtonian fluid, i.e., starch macromolecules are sufficiently distant so that no strong physical crosslinking occurs. Potentially formed bonds are easily broken under the action of mechanical forces and during testing they fail to form again. By analyzing the relationship between hydrophilic and hydrophobic interactions and the effects of secondary chemical bonds, it is noticed that the grafted macromolecule exhibits a specific behavior in the solution, which is reflected in viscosity and then in binding to the cotton yarn in the sizing process.

The viscosity stability of the solution of potential sizes was tested by heating to 85 °C for 7 h together with viscosity measurement every hour [26]. Table 5 shows the results of the viscosity tests for copolymer solutions and yarn types. It is noticeable that when the size concentration drops, the viscosity decreases, as expected. The effect of the copolymer solution temperature on viscosity is evident. The viscosity of the copolymer solution starting at 40 °C first increases by increasing the temperature to 60 °C, and then the viscosity decreases by further increasing the temperature to 85 °C. Namely, by increasing the temperature, the granules of the grafted starch swell, then disintegrate, and the structure of starch macromolecules weakens. The appearance of the so-called gelling process (swelling of starch, increasing density) is characteristic for each type of starch-copolymer and usually ranges from 50–70 °C [47].

The grafted products have a higher viscosity than a non-grafted hydrolyzed product, which is indicative of the good stability of potential sizes. By increasing the viscosity the surface size pick-up is higher, while in the case of low viscosity the size pick-up is lower on the yarn surface, but more uniform and higher in the yarn interior, which is an additional essential requirement for the sizing process. Higher size temperature allows a faster movement of size molecules, and hence a higher degree of overcoming adhesion forces and better adsorption of the size to the fibers in the yarn interior.

### 3.3. Testing of Sized Yarn

#### 3.3.1. Size Pick-Up

Yarn sizing was performed according to the designations in Table 6. One copolymer sample for each used copolymer showing the best results according to the analyses of hydrolysis and copolymerization (in Table 3 as bold values) was taken for sizing. By increasing the concentration of the sizing agent the size pick-up rises as expected. The size pick-up of the copolymer is slightly higher than the size pick-up of the hydrolyzed starch at the same concentration. It can be claimed that copolymer HS-AA shows the highest size pick-up. The ply yarn adsorbs or bonds more sizing agent in relation to finer yarns which is associated with higher concentration, higher volume, looser structure (yarn porosity) and with lower yarn twist. The size of lower concentration penetrates the structure—yarn depth—more easily, forms adhesive layers bonding the fibers together, while higher viscosity reduces the inner presence and increases surface pick-up. By analyzing the obtained results, it can be noticed that they coincide with the results reported in the literature [48,49,50,51].

#### 3.3.2. Breaking Strength and Elongation at Break

The test results of some mechanical properties, Table 7, reveal that after sizing there was an increase in the breaking forces of all the analyzed yarns, meaning that one of the basic tasks of the warp sizing processes was accomplished d. One of the tasks of the sizing process is that the breaking forces of the warp yarns are “sufficiently” higher than the maximum warp stretching in the weaving process.

Yarns sized with starch with MAA monomer achieves slightly better results of breaking force than yarns sized with starch with AA monomer. This indicates to more uniform distribution and appropriate adhesion of this starch type (with MAA monomer), both on the yarn surface and in the yarn interior. The consequence of sizing is a reduction in elongation at break of all yarns, probably because the yarn became encapsulated and trapped by the size coating, making it less elastic.

This reduction in breaking elongation of yarn is a shortcoming of sizing, whereby it is extremely significant that result variations are as low as possible which is manifested in this study. It is interesting to mention that breaking elongation of yarn sized with sizing agents with MAA and AA monomers hasn’t decreased a lot, which is convenient for practical use.

#### 3.3.3. Yarn Abrasion

**Figure 3:**
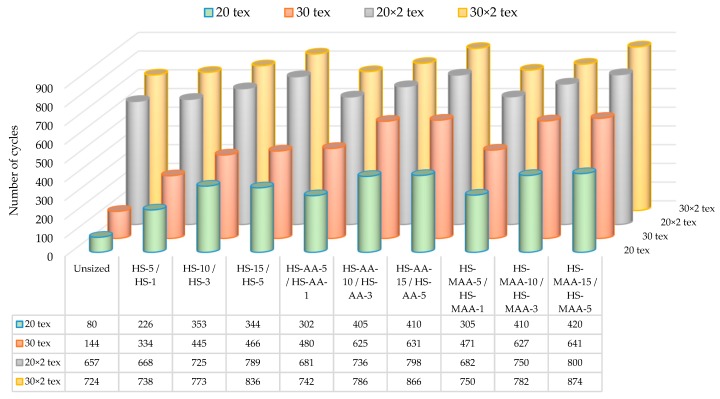
Yarn abrasion resistance during the sizing process.

Yarn abrasion resistance is one of the basic properties in weaving whereby the number of yarn breaks during weaving or weaving machine efficiency can be predicted, Figure 3 [52,53].

By applying the size to the yarn all tested samples of the sized yarn increased abrasion resistance or the number of abrasion cycles up to breakage.

Ply yarns (20 × 2 tex and 30 × 2 tex) has greater abrasion resistance before and after sizing process. The tendency to increase the abrasion resistance has a single yarns (20 tex and 30 tex) indicating the justification of conducting sizing process for this yarn. Yarns sized with hydrolyzed starch HS-MAA has a higher abrasion resistance increase compared to yarn sized with hydrolysed starch HS-AA starch.

Grafted starch proved to be an efficient product with regard to abrasion resistance. The reasons for such results of polymer chains in the starch macromolecular structure can be sought in its hydrophobicity. This fact can be the reason why the size softens or plasticizes, why fibers do not stick together or why some kind of yarn lubrication is achieved [54].

#### 3.3.4. Yarn Hairiness

**Figure 4:**
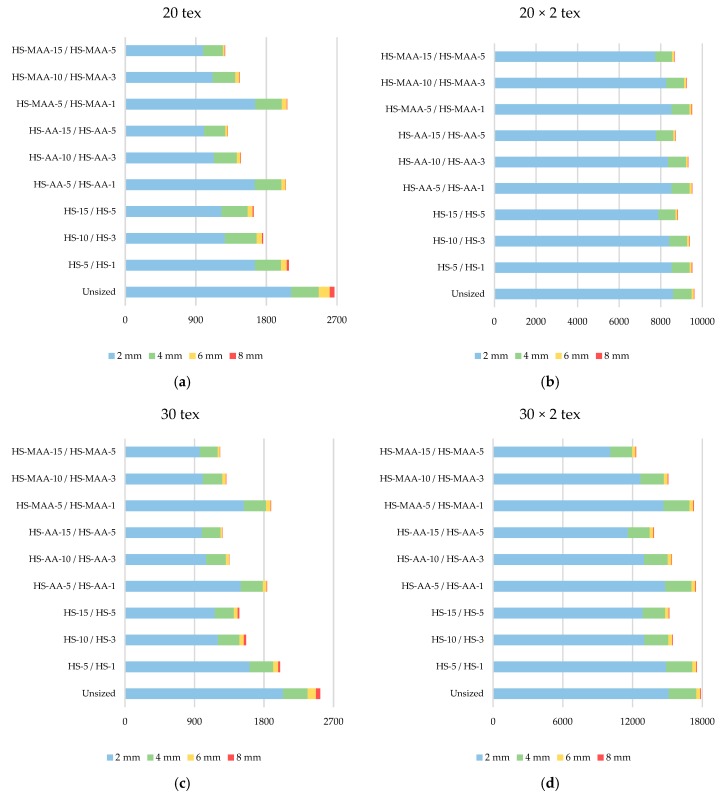
Hairiness of the yarn in the sizing process: (**a**) 20 tex single yarn, (**b**) 30 tex single yarn, (**c**) 20 × 2 tex ply yarn, (**d**) 30 × 2 tex ply yarn.

Hairiness can be defined as the state of migrated fiber ends and fiber loops pushed to the surface of the yarn body, not adhering to it. Some fibers have one of their ends fixed inside the yarn while others protrude under the influence of mechanical and geometrical causes. The intensity of yarn hairiness depends on the spinning method, degree of fiber parallelization, fiber types and lengths, yarn twist and other causes. One of the goals of sizing is to reduce yarn hairiness which negatively affects yarn abrasion in contact with the elements of the weaving machine. Yarn hairiness test results are shown in Figure 4.

The largest part of the protruding fibers are 2 mm long up to the yarn body. After sizing, the yarn hairiness (according to the measuring ranges) decreases, which is also one of the basic objectives of the sizing process. From the obtained results it is evident that sizing masses with higher concentrations have a better effect, i.e., the number of protruding fibers according to measuring ranges drastically decreases, even more in single then in ply yarns. Greater lengths of the protruding fibers cause thread entanglements and more yarn breakages in weaving. By observing the obtained results it is evident that the best effects of sizing in terms of hairiness reduction were achieved by using the HS-MAA-15/HS-MAA-5, for even more then 50% in single yarn and around 30% in plied yarn 30 × 2 tex, while this maximum reduction in yarn of finesses 20 × 2 tex is just 10%. The weakest effect on hairiness reduction was achieved with HS-5/HS-1 for all tested samples.

### 3.4. Degree of Desizing

Desizing was performed in two ways: by using ultrasound and washing off (Table 8). Hydrolyzed starch shows slightly lower results of desizing, by using washing and ultrasound. Hydrolyzed samples achieve a higher degree of desizing by using ultrasound. The best results of desizing by washing for the single yarn amount to 91% for hydrolyzed starches: HS-AA-5, HS-MAA-5 and FOR HS-MAA-10. In the case of the ply yarn the highest degree of desizing by washing amounts to 92% for HA-MAA-1.

The use of ultrasound in washing, as an additional source of highly usable energy, brings significant improvements, i.e., the removal of the size material from the warp yarn is more intensive. The best values of removing the size material from the single yarn by using ultrasound amount to 97% for starch HS-MAA-1 and in the case of the ply yarn the degree of desizing amounts also to 97% when using the same starch HS-MAA-1.

Sizing agents are major polluters of wastewaters in the textile industry due to great amounts of acids, oxidants, enzymes, bacteria etc. The results of the analysis of waste water after desizing are shown in Table 9. According to the results a higher temperature of waste water is noticeable in comparison to the limit value, which is not alarming, if the fact is taken into account that waste water can cool down and heat can be recovered; pH is in the range of limit values, the residue after evaporation is noticeable, Chemical Oxygen Demand (COD) and Biochemical Oxygen Demand (BOD5) have higher values than the limit values, which was expected, because no purification was carried out; COD/BOD5 ratio is a parameter, which is not prescribed by the standard, but is one of the most important ones because it indicates the biodegradability of waste water components irrespective of dilution. The obtained value of the ratio between biological and chemical oxygen demand (0.45) falls into the range from 0.2 to 0.5, indicating partial biodegradability.

### 3.5. Statistical Analysis of Individual Research Results

The analysis of individual research results from the point of view of statistical modelling and event prediction is of great importance in the analyses of individual yarn parameters that are affected by the sizing process.

This analysis deals with the parameter yarn breaking force as one of the most significant yarn parameters. It is one of the main reasons for sizing in order to improve yarn strength. The subject of research was interrelationship between breaking force (dependent variable) and the following parameters: degree of size pick-up, yarn hairiness, yarn count and yarn twist – independent variables. According to the basic yarn data and test results (Table 6 and Table 7) the equation of dependency of variables according to Table 10 is:

Yarn strength = 770.73 − 0.55 × number of twists + 2.40 × yarn count + 8.11 × degree of sizing + 0.0067 × hairiness index (2 mm)(8)

If one example of predicting the value of variable Yarn strength is taken for 20 tex yarn sized with AA copolymer and hydrolyzed potato starch (HS-AA-5), it is:

Yarn strength (predicted value) = 770.73 − 0.55 × 890 + 2.40 × 20 + 8.11 × 5.0 + 0.0067 × 2117(9)

Consequently, yarn strength (predicted value) = 383.96 cN.

Actual measured value of this variable is: Yarn strength (measured value) = 380.00 cN.

With a probability of 95% it is claimed that a single sized yarn has the value of variable Yarn strength of 383.96 cN. Practically, each value of the variable Yarn strength can be described with this model (coefficient of determination = 0.991). All variables contribute to the model, which is verified statistically (Prob > |t| = 7.43 × 10^−12^, 0.01828, 3.93 × 10^−13^ or 0.0022 < 0.05).

Table 11 determines more relevant parameters that indicate the efficiency and validity of multiple regression model. The correlation coefficient (R Value) and the coefficient of determination (R-Square (COD)) have almost maximum values, which confirms a high correlation between the criteria and the set of predictive variables. The standard error of estimate (Root-MSE (SD)), as a standard deviation of the residual (prediction error), clarifies the arrangements of the regression line with the data.

Analysis of variance for multiple regression indicates a statistically significant F Value (Prob > F 0 < 0.05), Table 12 There is a statistically significant linear correlation of the key variable—criterion variable and its predictors—input variable.

## 4. Conclusions

Hydrolyzed and grafted starches showed different properties, depending on the type of initiator used, but also on monomers. Based on the research methods used, mathematical and statistical analyses, it can be concluded:

Grafting with vinyl monomers depends on the used initiators. KPS initiator was the most efficient in grafting methacrylic acid onto hydrolyzed starch while AIBN initiator was very efficient in grafting of acrylic acid. The most important measurements in the synthesis of copolymers, yield of hydrolysis and grafting, grafting percentage and efficiency, conversion of monomer into polymer, as well as FTIR analysis confirmed that these are new starch and vinyl monomer products. All grafted products increase viscosity in comparison to non-grafted hydrolyzed starch, which is indicative of the good stability of a potential product. It is evident that the changes in temperature of the copolymer solution affects the viscosity. By increasing the concentration of starch, the size pick-up ratio also increases, and this is somewhat higher for polymers than for hydrolyzed starch, while the HS-AA copolymer is particularly emphasized. Ply yarns adsorb or bind to themselves more sizing agents in all cases in comparison to single yarns, which is related to higher volume, looser structure (yarn porosity) and smaller number of twists. Products with MAA copolymer achieve the best results with regard to breaking force for all types of yarn, which indicates their more uniform distribution within the yarn and good adhesion both over the surface and in the interior of yarn. A consequence of sizing is a reduction in the elongation at break of yarn, while products with MAA copolymer retain the elongation values which are approximate to the values of the unprocessed sample. By applying the size to the yarn, all tested samples of the sized yarn increased abrasion resistance or the number of abrasion cycles up to breakage. Grafted starch showed good resistance to abrasion. The best effects of sizing in terms of hairiness reduction were achieved with HS-MAA-15/HS-MAA-5, while the weakest effect was achieved with HS-5/HS-1, for all tested samples. In the desizing process by washing, as the most effective starches proved to be HS-AA-5, HS-MAA-5 and FOR HS-MAA-10 for single yarns and HA-MAA-1 for ply yarns. In the case of the desizing process using ultrasound, the starch HS-MAA-1 proved to be the easiest to remove from both single and ply yarns. 

The greatest advantage of polymers from an ecological point of view is manifested as biodegradability. The analysis results of the wastewater after desizing of the samples are promising for all tested parameters, where the obtained values of the ratio between biological and chemical oxygen demand belong to the range of values that indicate partial biodegradability. It is an advantage that the grafted starches do not disintegrate in sizing, but dissolve in water which allows recycling.

By analysis of the sized yarn it can be ascertained that synthesized starches yielded good results with regard to improving breaking force, abrasion resistance and reducing yarn hairiness. Statistical analyses of individual parameters and determining the finite models revealed their interrelationship, and thus the complexity of research. Careful selection of the synthesis process of the active agent, valid combination of modified sizing agents as well as the optimal choice of temperature-time regime make it possible to obtain an appropriate size paste composition and processing method which will enable uniform yarn sizing.

Based on the extensive analyses carried out, it can be concluded that economic, qualitative and environmental cost-effectiveness supports the reuse of natural starches in the sizing process, but only by synthesizing and grafting with appropriate initiators that significantly improve their properties. Further research will focus on the gradual reuse of natural synthesized starches, first as a mixture with synthetic starches, and then until their complete replacement.

## Figures and Tables

**Table 1:** Designations of the obtained copolymers, their meaning and recipe content per yarn types.

Copolymer Designation	Meaning of Designations	Graft Schemes
HS	Hydrolyzed starch	-
HS-AA-AIBN	Hydrolyzed starch grafted with acrylic acid, initiator azobisisobutyronitrile	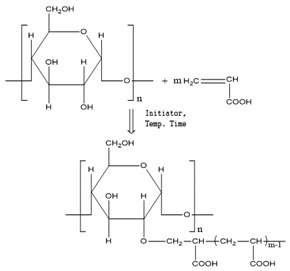
HS-AA-KPS	Hydrolyzed starch grafted with acrylic acid, initiator potassium persulfate	
HS-AA-BPO	Hydrolyzed starch grafted with acrylic acid, initiator benzoyl peroxide	
HS-MAA-AIBN	Hydrolyzed starch grafted with methacrylic acid, initiator azobisisobutyronitrile	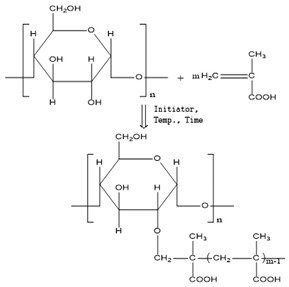
HS-MAA-KPS	Hydrolyzed starch grafted with methacrylic acid, initiator potassium persulfate	
HS-MAA-BPO	Hydrolyzed starch grafted with methacrylic acid, initiator benzoyl peroxide	

**Table 2:** Molar weights (g/mol) and degree of polydispersity of HS and copolymer depending on the initiator.

Sample	Elution Time 1.6–4.2 min				Elution Time 4.3–6.5 min			
	M_n_	M_w_	M_z_	D	M_n_	M_w_	M_z_	D
HS-AA-AIBN	1.40 × 10^6^	1.98 × 10^6^	2.73 × 10^6^	1.41	2.89 × 10^3^	3.64 × 10^3^	5.71 × 10^3^	1.26
HS-AA-BPO	1.33 × 10^6^	2.01 × 10^6^	2.88 × 10^6^	1.50	2.85 × 10^3^	3.47 × 10^3^	5.56 × 10^3^	1.22
HS-AA-KPS	1.06 × 10^6^	1.77 × 10^6^	2.72 × 10^6^	1.67	2.52 × 10^3^	3.35 × 10^3^	5.50 × 10^3^	1.33
HS	7.40 × 10^5^	1.32 × 10^6^	2.53 × 10^6^	1.77	1.59 × 10^3^	3.34 × 10^3^	5.49 × 10^3^	2.10
HS-MAA-AIBN	7.81 × 10^5^	2.16 × 10^6^	3.81 × 10^6^	2,76	1.76 × 10^3^	3.44 × 10^3^	5.74 × 10^3^	1.95
HS-MAA-BPO	7.59 × 10^5^	1.40 × 10^6^	2.89 × 10^6^	1.84	1.73 × 10^3^	3.40 × 10^3^	5.70 × 10^3^	1.96
HS-MAA-KPS	9.14 × 10^5^	1.54 × 10^6^	2.69 × 10^6^	1.69	2.03 × 10^3^	3.53 × 10^3^	5.54 × 10^3^	1.74
HS	7.44 × 10^5^	1.32 × 10^6^	2.53 × 10^6^	1.77	1.59 × 10^3^	3.34 × 10^3^	5.49 × 10^3^	2.10

**Table 3:** FTIR spectra of the hydrolyzed and grafted starch per individual types.

Samples		Ysh (%)	Yg (%)	Pg (%)	Pge (%)	Cmp (%)
Hydrolyzed starch	HS	84.62	-	-	-	-
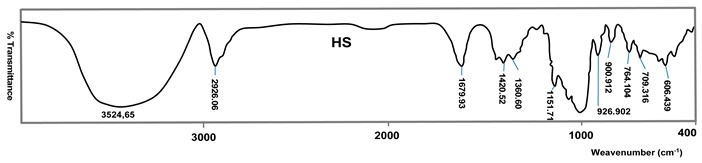						
Copolymer of acrylic acid and potato starch	HS-AK-BP	-	**87.50**	**27.27**	**60.98**	**98.40**
	HS-AK-AIBN	-	86.25 ^a^	25.45 ^a^	56.68 ^a^	98.80 ^b^
	HS-AK-KPS	-	86.88 ^b^	26.36 ^b^	59.06 ^b^	98.20 ^b^
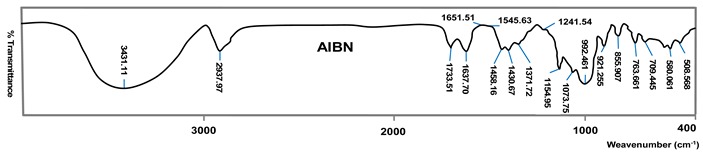						
Copolymer of methacrylic acid and potato starch	HS-MK-BP	-	87.50 ^a^	27.27 ^a^	60.98 ^a^	98.40 ^b^
	HS-MK-AIBN	-	**89.38**	**30.00**	**67.21**	**98.20**
	HS-MK-KPS	-	88.75 ^a^	29.09 ^b^	64.78 ^a^	98.80 ^b^
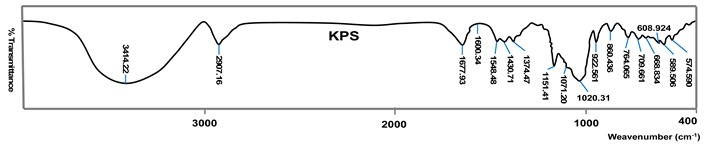						

Where: Ysh—yield of starch hydrolysis (%), Yg—graft yield (%), Pg—grafting percentage (%), Pge—percentage of graft efficacy (%), Cmp—conversion of monomer to polymer (%), ^a^—for significance level 0.05, mean value of the group sample is significantly different from the hypothetical mean value, ^b^—for significance level 0.05, mean value of the group sample is not significantly different from the hypothetical mean value.

**Table 4:** HPLC chromatograms and single values from the chromatogram per samples and grafting initiators.

Type of Sample		HPLC Chromatogram	RV (min)	PP (mAU⋅s)	VP (nRIU)
Copolymer of acrylic acid and potato starch	HS-AA-AIBN	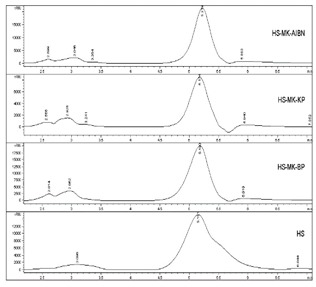	5.219	464,623.0	21,420.0
	HS-AA-KPS		5.180	524,846.4	20,374.0
	HS-AA-BPO		5.174	253,251.5	9413.4
Hydrolyzed potato starch	HS		5.151	611,369.3	15,321.5
Hydrolyzed potato starch	HS	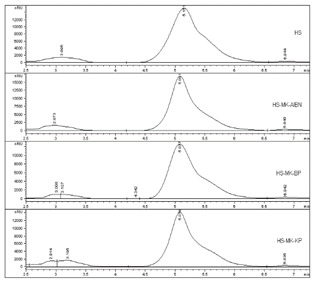	5.081	557,399.9	17,076.9
Copolymer of methacrylic acid and potato starch	HS-MAA-AIBN		5.077	459,107.5	12,664.9
	HS-MAA-BP		5.078	490,271.6	14,286.1
	HS-MAA-KPS		5.151	611,369.3	15,321.5

Where: RV—retention time or the time required to elute the copolymer or hydrolysed starch (min), PP—peak area (mAU-s), VP—peak height (nRIU).

**Table 5:** Viscosity of the copolymer solution in different concentrations according to yarn types.

Starch Designation (Single/Plied Yarn)	Viscosity, η (mPa·s)					
	Single Yarn			Ply Yarn		
	Temperature (°C)					
	40	60	85	40	60	85
HS-5/HS-1	38.8	57.1	53.9	11.5	16.3	14.9
HS-10/HS-3	46.2	65.2	61.9	22.6	37.1	35.5
HS-15/HS-5	53.6	71.8	70.2	38.8	57.1	53.9
HS-AA-5/HS-AA-1	41.2	59.5	55.5	12.4	18.1	16.5
HS-AA-10/HS-AA-3	53.2	61.1	60.9	24.5	37.9	36.1
HS-AA-15/HS-AA-5	63.5	69.3	68.6	41.2	59.5	55.5
HS-MAA-5/HS-MAA-1	41.0	58.1	52.3	12.5	17.5	17.0
HS-MAA-10/HS-MAA-3	52.9	63.4	60.1	24.4	37.3	36.6
HS-MAA-15/HS-MAA-5	63.2	70.1	68.4	41.0	58.1	52.3

Where: designations 1, 3, 5, (e.g.: HS-1, HS-3, HS-5 …) and 5, 10, 15 (e.g.: HS-5, HS-10, HS-15 …) indicate the copolymer content in the aqueous sizing solution according to the recipe.

**Table 6:** Size pick-up on the yarn sized with grafted hydrolyzed starch.

Starch Designation (Single/Plied Yarn)	Size Pick-Up (%)			
	Single Yarn		Ply Yarn	
	20 tex	30 tex	20 × 2 tex	30 × 2 tex
HS-5/HS-1	4.0	4.9	0.9	1.1
HS-10/HS-3	7.8	8.9	2.2	2.6
HS-15/HS-5	13.8	15.6	4.0	4.2
HS-AA-5/HS-AA-1	5.0	6.4	1.2	1.4
HS-AA-10/HS-AA-3	9.1	10.5	3.2	3.6
HS-AA-15/HS-AA-5	16.1	18.2	5.3	6.7
HS-MAA-5/HS-MAA-1	4.8	6.2	1.0	1.2
HS-MAA-10/HS-MAA-3	8,8	10.3	2.8	3.0
HS-MAA-15/HS-MAA-5	14.8	17.0	4.9	6.5

**Table 7:** Breaking forces and elongations at break of the yarn per counts and starch types.

Starch Designation	Yarn Fineness															
	20 tex				30 tex				20 × 2 tex				30 × 2 tex			
	F_20_ (cN)	CV (%)	Ɛ_20_ (%)	CV (%)	F_30_ (cN)	CV (%)	Ɛ_30_ (%)	CV (%)	F_20 × 2_ (cN)	CV (%)	Ɛ_20 × 2_ (%)	CV (%)	F_20 × 2_ (cN)	CV (%)	Ɛ_30 × 2_ (%)	CV (%)
Before sizing	330	8.1	4.1	9.1	459	7.4	5.4	7.1	650	4.4	6.3	6.8	824	5.3	5.1	5.5
HS-5/HS-1	349	9.2	2.9	8.8	478	6.6	3.8	6.5	665	4.8	5.5	7.5	835	4.5	4.6	6.2
HS-10/HS-3	410	8.2	2.8	7.5	546	6.1	3.7	6.3	675	2.8	5.4	8.3	852	4.4	4.5	7.3
HS-15/HS-5	445	7.8	2.7	7.9	574	7.2	3.5	5.5	688	5.2	5.3	9.6	877	3.5	4.3	5.8
HS-AA-5/ HS-AA-1	380	7.7	3.2	8.5	490	7.8	4.2	7.4	670	3.9	6.0	4.5	840	3.7	4.6	5.8
HS-AA-10/ HS-AA-3	438	7.5	3.2	8.2	560	7.9	4.1	7.6	682	3.1	5.7	6.9	840	3.9	4.4	6.5
HS-AA-15/ HS-AA-5	452	7.3	3.0	7.9	591	7.9	4.1	7.8	698	6.0	5.7	5.7	882	5.3	4.2	6.0
HS-MAA-5/ HS-MAA-1	380	8.6	3.4	8.5	495	8.5	4.4	8.0	674	5.7	6.0	8.9	840	6.4	4.9	9.6
HS-MAA-10/ HS-MAA-3	441	8.3	3.4	7.8	582	8.3	4.4	8.1	685	4.2	6.0	9.5	857	4.5	4.8	7.5
HS-MAA-15/ HS-MAA-5	460	8.1	3.3	7.9	595	8.0	4.2	8.9	699	5.3	5.7	9.9	900	5.7	4.5	8.9

Where: F_20,30,20×2,30×2_—breaking force according to yarn designations (cN), Ɛ_20,30,20×2,30×2_—elongation at break according to yarn designations (%), CV—variation coefficient (%).

**Table 8:** Degree of yarn desizing by using washing/ultrasound off.

Recipe Designation	Degree of Yarn Desizing: Using Washing/Ultrasound (%)			
	20 tex	30 tex	20 × 2 tex	30 × 2 tex
HS-5/HS-1	80/90	70/78	85/90	75/83
HS-10/HS-3	75/85	66/74	80/85	70/76
HS-15/HS-5	72/82	65/72	77/83	65/70
HS-AA-5/HS-AA-1	91/94	90/94	90/95	90/95
HS-AA-10/HS-AA-3	90/93	88/90	89/94	88/91
HS-AA-15/HS-AA-5	84/89	81/84	85/90	82/86
HS-MAA-5/HS-MAA-1	90/96	91/97	91/96	92/97
HS-MAA-10/HS-MAA-3	89/93	91/95	89/94	90/94
HS-MAA-15/HS-MAA-5	85/89	87/91	85/90	88/92

**Table 9:** Results of the water analysis after desizing and biodegradability.

Parameter	Standard	Units	Analysis Result	Limit Value
Appearance/color	EN ISO 7887:2001	-	murky	clear/colorless
Temperature		°C	60	30
pH	ISO 10523:1998	-	8	6.5–9
Residue after evaporation	ISO 3696	mg/L	250	-
COD	ISO 6060:1994	mg/L O_2_	8,484 (natural starch: 10,000–20,000)	200
BOD_5_	EN 1899-1:2009	mg/L O_2_	3812 (natural starch: 5000–10,000)	30
BOD_5_/COD	-	-	0.45	<0.2 non-biodegradable 0.2–0.5 partially biodegradable >0.5 biodegradable

**Table 10:** Values of the coefficient of regression model of variable Breaking force.

Model Parameters	Value	Standard Error	*t*-Value	Prob > |t|	95% LCL	95% UCL
Intercept	770.73	61.34	12.56	1.58 × 10^−14^	646.19	895.26
Number of twists	−0.55	0.055	−10.05	7.43 × 10^−12^	−0.67	−0.44
Yarn count	2.40	0.97	2.47	0.01828	0.43	4.38
Size pick-up (20 tex)	8.11	0.72	11.21	3.93 × 10^−13^	6.64	9.58
Hairiness (2 mm)	0.0067	0.0020	3.30	0.0022	0.0026	0.0108

**Table 11:** Parameters of efficiency of the regression model of variable Yarn strength.

Parameters	Yarn Strength
Number of Points	40
Degrees of Freedom	35
Residual Sum of Squares	10,274.24
R Value	0.995
R-Square (COD)	0.991
Adj. R-Square	0.9899
Root-MSE (SD)	17.133

**Table 12:** ANOVA for multiple regression of variable Yarn strength.

	DF	Sum of Squares	Mean Square	F Value	Prob > F
Model	4	1.12 × 10^6^	280,753.03	956.41	0
Error	35	10,274.25	293.55		
Total	39	1.13 × 10^6^			
